# Antimicrobial, antioxidant, anti-inflammatory, and cytotoxic activities of *Cordyceps militaris* spent substrate

**DOI:** 10.1371/journal.pone.0291363

**Published:** 2023-09-08

**Authors:** Danyu Zhang, Qingjiu Tang, Xianzhe He, Yipeng Wang, Guangyong Zhu, Ling Yu

**Affiliations:** 1 School of Perfume and Aroma Technology, Shanghai Institute of Technology, Shanghai, China; 2 Institute of Edible Fungi, Shanghai Academy of Agricultural Sciences, Shanghai, China; National Botanical Research Institute CSIR, INDIA

## Abstract

*Cordyceps militaris* is a medicinal mushroom and has been extensively used as a traditional medicine in East Asia. After the chrysalis seeds are matured and harvested, the spent substrate of *C*. *militaris* still contains active ingredients but is usually discarded as waste. This study aimed to determine the antioxidant and anti-inflammatory activities of *C*. *militaris* spent substrate extract and its inhibitory activity on the *Malassezia* commensal yeasts that can cause dandruff and seborrheic dermatitis. Active substances in the spent substrate of *C*. *militaris* were extracted using a hot water extraction method and were used for the determination of antioxidant activity by measuring their ability to scavenge 2,2-diphenyl-1-picrylhydrazyl (DPPH), hydroxyl radicals, hydrogen peroxide, and superoxide anions. The ability to inhibit *Malassezia* was analyzed using the broth microdilution method, and the reparative effect on oxidative damage in HaCaT cells was measured using *in vitro* cell analysis. Respiratory burst evaluation was used to determine the anti-inflammatory capacity of extracts. Analysis of the *Malassezia*-inhibiting activity of the extracts showed that the minimum inhibitory concentration was 6.25 mg/mL. The half maximal inhibitory concentration (IC50) values of DPPH, O_2_^-^, H_2_O_2_ and OH^-^ were 3.845 mg/mL, 2.673 mg/mL, 0.037 mg/mL and 0.046 mg/mL, respectively. In the concentration range of 2 to 50%, the extract was non-toxic to cells and was able to protect HaCaT cells from H_2_O_2_ damage. When the volume fraction of the extract was 20.96%, its anti-inflammatory ability reached 50%. These results demonstrated that the extract may be a safe and efficacious source for pharmaceutical or cosmetic applications, with *Malassezia*-inhibiting, antioxidant and anti-inflammatory activities.

## 1. Introduction

*Malassezia* spp. are commensal yeasts that can cause cutaneous ailments such as dandruff and seborrheic dermatitis. *Malassezia* secretes lipases and hydrolases that act on human sebum and cause the release of diglycerides and unsaturated and saturated fatty acids on the scalp. Some of these unsaturated fatty acids penetrate the stratum corneum, inducing inflammation and enhancing abnormal keratinization [1]. Studies showed that certain natural materials from mushrooms [2], neem, licorice, pomegranate, and ghirtkumari [3] have antimicrobial activities and can be used as raw materials in skincare products.

The generation of free radicals or reactive oxygen species from the incomplete reduction of molecular oxygen during aerobic respiration is closely related to cellular damage. An excess of reactive oxygen species leads to severe oxidative stress, which leads to nucleic acid damage, polyunsaturated fatty acid oxidation in lipids, and amino acid oxidation in proteins [4]. The antioxidant properties of many phytochemicals (e.g., phenolic compounds) allow them to reduce reactive oxygen species production [5]. Mycelium extracts of some mushrooms reduce oxidative stress by increasing the activity of antioxidant enzymes [6]. Normal physiological processes require a balance between cellular processes that produce reactive oxygen species and antioxidant defense systems that remove them [7]. Due to their safety and widespread distribution, plant-based antioxidants have gained considerable attention in recent years. Studies have demonstrated that they are highly effective scavengers of a broad spectrum of oxidants and inhibitors of lipid peroxidation. In addition to antioxidants, antimicrobial agents have been reported in a variety of medicinal plants.

The cultivation substrate left after harvesting edible mushroom seed entities is edible mushroom bran, which consists of mycelium, straw, other crude fibers and fermentation metabolites. Edible mushroom bran contains high concentrations of carbohydrates, proteins, amino acids, vitamins, and trace elements. One kilogram of fresh mushrooms generates approximately 5 kg of spent mushroom substrate [8]. *Cordyceps militaris*, an entomopathogenic fungus belonging to the class Ascomycetes, is a valuable medicinal fungus and a modern rare herbal medicine in China [9]. Many bioactive molecules of nutraceutical interest, such as cordycepin(3’-deoxyadenosine), ergosterol, trehalose, mannitol, and several polysaccharides, nucleosides, and amino acids have been detected in or isolated from *C*. *militaris*, and these are thought to have potential antiaging, whitening, antitumor, anti-inflammatory, immunomodulatory, and blood glucose and cholesterol-lowering activities [10]. Of these, cordycepin is the main active constituent that is most widely studied for its medicinal value and nutraceutical potential. Cordycepin exhibits a wide range of beneficial health effects, such as antimicrobial [11], antioxidant, anti-inflammatory, immunomodulatory [12], and antitumor activities [13]. Almost all biologically active components are extracted from the fermented solution or fruit bodies, while the spent cultivation substrate used to cultivate the *C*. *militaris* fruit body is rarely used after harvest. Due to the advantage of the spent substrate in many applications, compared with the *C*. *militaris* fruit body, there is great potential for its scientific study and commercial value [14]. While the fruiting bodies and mycelium of *C*. *militaris* have been investigated, there is limited information on the biochemical properties of the spent substrate of *C*. *militaris* (bacteriostatic, antioxidant, anti-inflammatory, and cytotoxic activities).

In this study, we aimed to explore the bacteriostatic, antioxidant and anti-inflammatory effects of the spent substrate extract of *C*. *militaris* and to clarify whether cordycepin is the main *Malassezia* inhibitory component. Our results may provide new ideas for further investigation and development of *C*. *militaris* waste substrate and lay a solid foundation for its application in cosmetics and as therapeutic agent.

## 2. Materials and methods

### 2.1. Materials

Spent substrate was obtained from the Shanghai Academy of Agricultural Sciences (Shanghai, China). The dried spent substrate of *C*. *militaris* was ground into a fine powder using an 80-mesh and a high-speed grinder (DSY-9002, Yongkang Jiu shunying Trading Co., Ltd., China) and stored in a desiccator.

### 2.2. Reagents and cell lines

Methanol, cordycepin, 97% luminol, 2,2-diphenyl-1-picrylhydrazyl (DPPH) and phorbol 12-myristate 13- acetate (PMA) were purchased from Sigma (USA). Phenol, concentrated sulfuric acid, ethanol, trifluoroacetic acid, cuprous chloride, sodium dihydrogen phosphate, and disodium hydrogen phosphate were purchased from Sinopharm Chemical Reagent Co. Ltd (CN). NKA-II macroporous adsorption resin was purchased from Shanghai Yuanye Biotechnology Co., Ltd (CN). D101 macroporous adsorption resin was purchased from Cangzhou Baoen Adsorbent Material Technology Co. Ltd (CN). 30% hydrogen peroxide was from Shanghai Taopu Chemical Company, and o-phenanthroline was from Shanghai Jingdian Chemical Technology Co. Ltd (CN). AlamarBlue reagent was purchased from Thermo Scientific (USA). All the reagents were of analytical grade.

Mouse macrophage RAW 264.7 and immortalized human keratinocytes (HaCaT cells) were provided by the Institute of Edible Mushrooms, Shanghai Academy of Agricultural Sciences. *Malassezia* furfur ATCC 14521 was purchased from the China Industrial Microbial Strain Collection Management Center. Dulbecco’s modified Eagle’s medium (DMEM), high glucose DMEM, heat-inactivated fetal bovine serum, glutamine, penicillin, and streptomycin were purchased from Gibco (USA).

### 2.3. Preparation of extracts

The spent substrate of *C*. *militaris* was incubated with distilled water (1:20, w/v) at 100°C for 2 h, and the extract was collected by filtration. The material was vacuum filtered, and the precipitate was subjected to the same process. After two extractions, the aqueous filtrates were combined. The obtained extract was stored at −20°C until use. The extractions were performed in triplicates.

Different components of the extract were separated through adsorption on macroporous resin. Briefly, 80 g each of D101 and NKA-II macroporous adsorption resin were mixed with 200 mL of *C*. *militaris* spent extract and incubated for 8 h at 26°C on a shaker with protection from light. The resin and the adsorption supernatant fraction were then collected by filtration, and the macroporous adsorption resin was eluted with 95% ethanol to obtain the eluted fraction. Adsorption supernatants and ethanol-eluted fractions from each resin were lyophilized.

To test the antimicrobial effects of *C*. *militaris* spent extract, NKA-II macroporous adsorption resin was used to gradually elute the extract using 30%, 50%, 70%, and 90% ethanol, and the prepared components were lyophilized.

### 2.4. Characterization of the extract

Total sugar content was determined with the phenol-sulfuric acid method using glucose as the standard [15].

The content of cordycepin in the spent substrate extract of *C*. *militaris* was determined by a Waters high-performance liquid chromatography (HPLC) system (Waters 2695) [16] using the Venusil MP C18 column (250 mm × 4.60 mm, 5 μm) at room temperature. Chromatographic parameters were as follows: mobile phase, methanol-water (20:80); flow rate, 1 mL/min; UV detection wavelength, 254 nm; and injection volume, 10 μL. The prepared cordycepin solution was weighed and passed through a microporous filter membrane (0.22 μm), and then the sample was tested. The peak area Y was linearly regressed with the mass concentration X of cordycepin (mg/mL).

### 2.5. Antimicrobial evaluation

The antimicrobial evaluation was performed according to the American Clinical and Laboratory Standards Institute (CLSI) judgment criteria [17]. Briefly, 100 μL of *Malassezia* suspension (3 × 10^8^ CFU/ mL), 100 μL of the sample, and 10 μL of olive oil were added to each well of a 96-well plate. For the negative control group, 100 μL of sterile water was added instead of the sample. Ketoconazole (0.16 ug/mL) was used as a positive control for the *in vitro* inhibition test, and testing was performed in triplicate. The samples were incubated at 32°C for 48 h. Because *Malassezia* can only grow when floating on the surface of an oil layer, the naked eye observation method was used. The concentration of the *C*. *militaris* solid fermentation extract in the sample with complete fungal inhibition was used for the minimum bactericidal concentration (MBC) value, and the concentration of the extract in the sample with 50% fungal growth inhibition was taken as the minimum inhibitory concentration (MIC) value.

### 2.6. Antioxidant evaluation

The *in vitro* antioxidant potential of the spent substrate extract of *C*. *militaris* was evaluated by assessing its free radical quenching ability using DPPH, hydroxyl radical, hydrogen peroxide, and superoxide anion scavenging assays.

#### 2.6.1. DPPH radical scavenging assay

The DPPH radical scavenging activities of the spent substrate extract of *C*. *militaris* were measured as reported by Blois [18, 19], with some modifications. A volume of 0.2 mL of sample solution (sample group) or 80% ethanol (blank group) was mixed with 2.8 mL of a 0.1 mmol/L DPPH solution and incubated for 30 min in the dark at 25°C. The optical density (OD) value at 517 nm was measured using a microplate reader (Synergy HT, Bio-TEK, USA). Three replicate wells were used for each group and the average value was calculated. The formula for the DPPH clearance rate was as follows:

$$Scavenging\;rate(\%)=\frac{A0-A1}{A0}\times 100\%$$ (1)

where A1 is the absorbance value of the sample solution at 517 nm, and A0 is the absorbance value of the blank group at 517 nm.

#### 2.6.2. Superoxide anion scavenging assay

The superoxide anion scavenging activity of the spent substrate extract of *C*. *militaris* was assayed using an o-phenothrin-luminol system [20, 21]. A carbonate buffer solution (pH 10) at a concentration of 50 mmol/L and a luminol solution of 1 mmol/L were mixed well in a 2:1 ratio (working solution). In a 96-well plate, 150 μL of the working solution was then mixed with 10 μL of different concentrations of the spent substrate extract of *C*. *militaris*, and with 10 μL of a 0.625 mmol/L catechol solution. Chemiluminescence was evaluated with a luminometer (Clarity-2PC, Bio-TEK, USA). The reaction time was set for 1 s, and the data were recorded continuously for 30 s. The highest value A1 was obtained, and the peak control value was recorded as A0 with deionized water as the blank control. Three replicate wells were used in each group and the average value was calculated. The formula for the superoxide anion clearance rate was:

$$Scavenging\;rate(\%)=\frac{A0-A1}{A0}\times 100\%$$ (2)

#### 2.6.3. Hydroxyl radical scavenging assay

The hydroxyl radical scavenging activity was analyzed using a chemiluminescence method [22, 23]. A carbonate buffer solution (pH 8.5) with a concentration of 50 mmol/L and a luminol solution of 1 mmol/L was mixed at a ratio of 17:1 (working solution) away from light. For the assay, 10 μL of different concentrations of the spent substrate extract of *C*. *militaris*, 10 μL of a 6% H_2_O_2_ solution, 10 μL of a 1 mmol/L CuCl solution, 10 μL of a 1 mmol/L o-phenanthroline solution, and 150 μL of the working solution were added to a 96-well plate. The reaction time was set for 2 s. Chemiluminescence was measured and recorded continuously for 15 s to obtain the highest value, A1, with deionized water as blank control. The peak control value was measured and recorded as A0. Three replicate wells were used in each group, and the average value was calculated. The formula for hydroxyl radical clearance was:

$$Scavenging\;rate(\%)=\frac{A0-A1}{A0}\times 100\%$$ (3)

#### 2.6.4. Hydrogen peroxide scavenging assay

The H_2_O_2_-scavenging activities were analyzed using a chemiluminescence method [24]. A carbonate buffer solution (pH 9.5) with a concentration of 50 mmol/L and a luminol solution of 1 mmol/L were mixed at a ratio of 17:1 away from light (working solution). For the measurement, 10 μL of different concentrations of the spent substrate extract of *C*. *militaris*, 10 μL of a 6% H_2_O_2_ solution, and 150 μL of the luminol-carbonate system luminescence working solution were added to the a 96-well plate, and the chemiluminescence was measured. The reaction time was set to 30 s, and the experimental data were measured every 0.3 s to obtain the highest value, A1, with deionized water as a blank control. The peak control value was recorded as A0.

Three replicate wells were used for in each group and the average value was obtained. The formula for hydrogen peroxide anion clearance was:

$$Scavenging\;rate(\%)=\frac{A0-A1}{A0}\times 100\%$$ (4)

### 2.7. *In vitro* cellular analysis of the spent substrate extract of *C*. *militaris*

*In vitro* cell analysis of the spent substrate extract of *C*. *militaris* was performed to determine its protective and reparative effects on H_2_O_2_-damaged cultured human keratinocyte cells (HaCaT cells).

#### 2.7.1. Effects of the spent substrate extract of *C*. *militaris* on the activity of H_2_O_2_-damaged HaCaT cells

HaCaT cells were cultured in 5% CO_2_ at 37°C in regular DMEM supplemented with 10% heat-inactivated fetal bovine serum, glutamine (2 mM), penicillin (100 U/mL), and streptomycin (100 μg/mL). Cells were grown to 80% confluence, trypsinized, and centrifuged at 1000×*g* for 3 min (centrifuge 5415D, Eppendorf). The pellets were diluted to 1 × 10^5^/mL with a growth medium and transferred into 96-well cell culture plates at a concentration of 1 × 10^4^ cells/well, and 100 μL of culture medium with different concentrations of *C*. *militaris* spent extract were added. PBS was used as control. Cells were cultured in a CO_2_ incubator for 24 h. After 24 h, cell viability was assessed using AlamarBlue reagent. The cells were placed in the incubator for 6 h, and the resulting fluorescence was read on a plate reader (Synergy HT, Bio-TEK, USA) to detect cell viability and calculate cell proliferation rate. The calculation formula was

$$Proliferation\;rate(\%)=\left(1-\frac{{OD570}_{\left(sample\right)}\times 117.216-{OD600}_{\left(sample\right)}\times 80.586}{{OD570}_{\left(sample\right)}\times 117.216-{OD600}_{\left(control\right)}\times 80.586}\right)$$ (5)

#### 2.7.2. Establishment of an H_2_O_2_ oxidative stress model

Cells were cultured in a 96-well cell culture plate. The culture medium was replaced with 100 μL of medium containing six concentration gradients of H_2_O_2_ (range of 0.1–0.6 mmol/L) and cells were incubated for 24 h in a CO_2_ incubator. PBS was used as a control. Cells were stained with AlamarBlue reagent and incubated for 6 h in the CO_2_ incubator, at which time cell activity and proliferation rate were calculated. An H_2_O_2_ concentration leading to cell damage of 50% was selected as an indicator.

#### 2.7.3. Protective effect of the spent substrate extract of *C*. *militaris* on H_2_O_2_-damaged HaCaT cells

The spent substrate extract of *C*. *militaris* was diluted with sterile PBS to 50%, 15%, 8%, 4%, and 2% v/v. HaCaT cells were cultured overnight in a 96-well cell culture plate and incubated with the medium containing different volume fractions of the extract for 24 h. Cells were then incubated with a fresh culture medium containing the appropriate concentration of H_2_O_2_ for 24 h. PBS was used as control.

### 2.8. Determination of the anti-inflammatory capacity

RAW 264.7 cells were cultured in a 5% CO_2_ incubator at 37°C in high glucose DMEM supplemented with 10% heat-inactivated fetal bovine serum, glutamine (2 mM), penicillin (100 U/mL), and streptomycin (100 mg/mL). A respiratory burst assay [23] was used to test the anti-inflammatory capacity of chrysalis solid fermentation extracts by incubating 180 μL of 5 × 10^5^ RAW 264.7 cells/mL, 50 μL of the luminol working solution, 2.5 μL (2 × 10^−2^ mg/mL) of PMA, and 10 μL of chrysalis solid fermentation extracts. The reaction was performed for 40 min, and data were measured every 1 min to obtain the peak A1. PBS was substituted for the sample to obtain the peak A0. Three replicate wells were used for each group and the average value was obtained. The formula for hydrogen peroxide anion clearance was:

$$Scavenging\;rate(\%)=\frac{A0-A1}{A0}\times 100\%$$ (6)

### 2.9. Statistical analysis

Excel 2007 software was used to process the data, and statistical analysis was performed using SPSS 22 software. Triplicate studies were performed, and the results were expressed as the mean ± standard deviation. P < 0.05 was considered statistically significant.

## 3. Results and discussion

### 3.1. Active matter content of the extract

**Fig 1:**
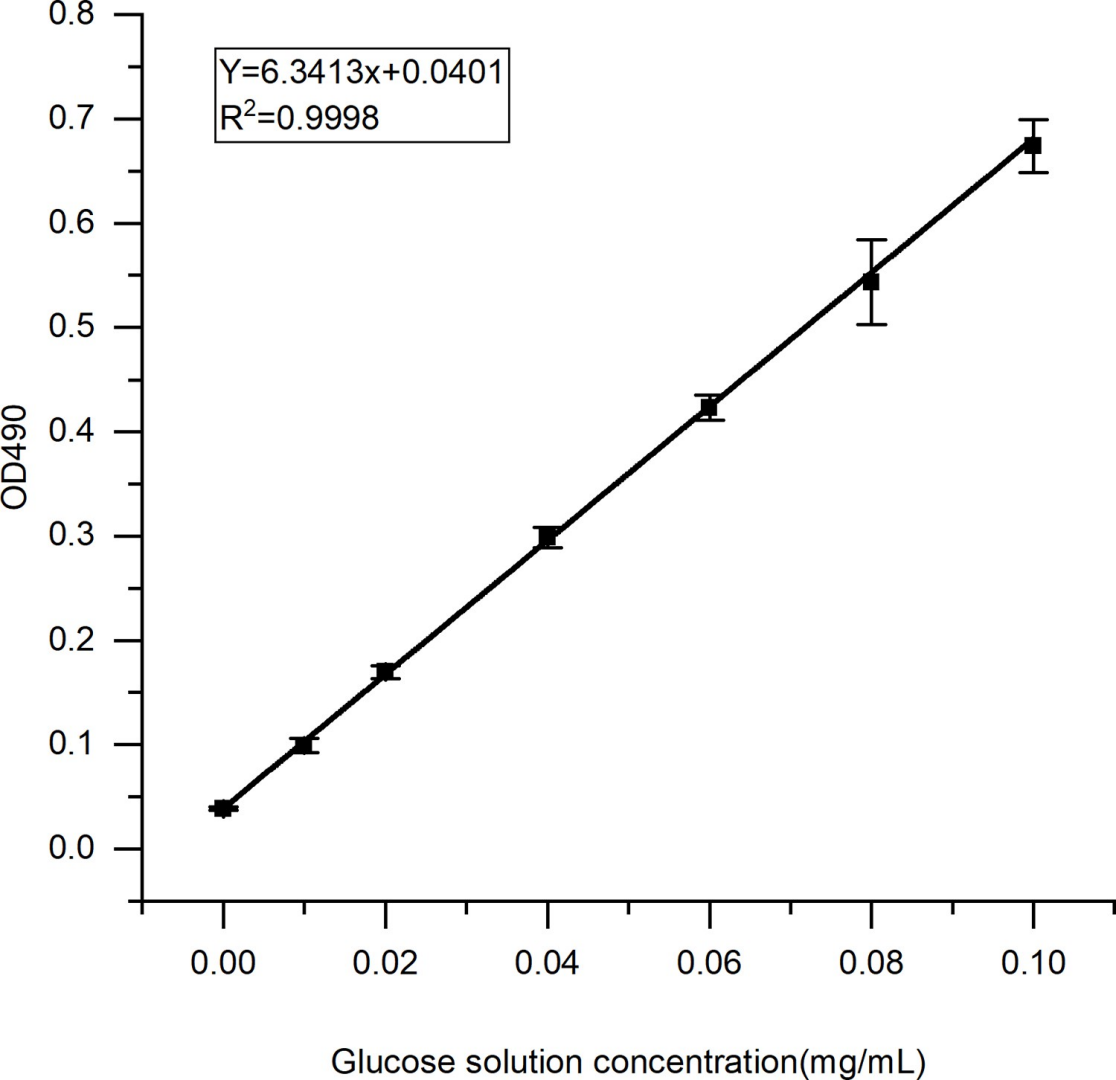
Standard curve for total sugar content determined using the phenol-sulfuric acid method.

The total sugar content of *C*. *militaris* spent extract was determined using the phenol-sulfuric acid method. The total sugar regression equation was as follows:: Y = 6.3413X + 0.0401; R^2^ = 0.9998. (p = 0.010 < 0.05) (Fig 1).

**Fig 2:**
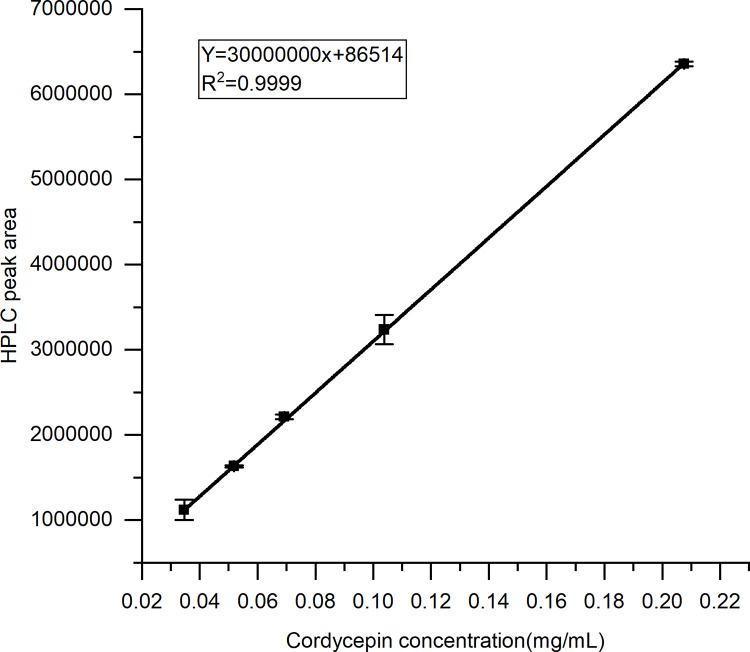
Cordycepin mass concentration and HPLC peak area standard curve.

The cordycepin content was determined using HPLC and showed a good correlation with the peak area in the range of approximately 0.04–0.2 mg/mL. The regression equation was as follows: Y = 3 × 10^7^X + 86,514; R^2^ = 0.9999 (p = 0.035 < 0.05) (Fig 2).

The extraction rate of cordycepin was 0.846 ± 0.0058%, and the extraction rate of the total sugar was 5.829 ± 0.087%.

### 3.2. Analysis of the antimicrobial ability and main active ingredients of the extract

#### 3.2.1. Inhibitory effect of different fractions on *Malassezia*

The ability of the *C*. *militaris* spent substrate extract to inhibit *Malassezia* was determined using the broth microdilution method. The results showed that the raw extract had an MIC value of 6.25 mg/mL and an MBC value of 12.5 mg/mL at the concentration of the raw drug.

As summarized in Table 1, the adsorption separation of the inhibitory components was greater when NKA-II macroporous adsorption resin was used, and the inhibition ability of the NKA-II 95% ethanol-eluted fraction was the highest. The inhibition ability of the NKA-II adsorption supernatant was relatively poor, indicating that the main inhibitory components in the extract of the spent substrate of *C*. *militaris* were effectively adsorbed and enriched by this resin. The adsorption effect of the D101 macroporous resin was relatively weak, and the adsorption effect of the supernatant was greater than that of the 95% ethanol eluted-fraction, suggesting that the adsorption effect of the D101 macroporous resin on the effective antimicrobial components was not as high as that of the NKA-II macroporous resin. NKA-II, therefore, was used for all the subsequent experiments. As shown in Table 2, 30% ethanol was used to elute most of the effective inhibitory components adsorbed by NKA-II resin. The MIC of this fraction was 0.75 mg/mL, the MBC was 0.75 mg/mL, and the inhibition of *Malassezia* was slightly less effective than that of the 95% ethanol elution fraction. The abilities of 50%, 70%, and 90% ethanol elution fractions to inhibit *Malassezia* were relatively similar, and therefore, it was presumed that the difference in the content of the effective inhibitory components was not significant.

**Table 1:** MIC and MBC results of samples separated using different macroporous adsorption resins against *Malassezia*.

Sample	Sample concentration (mg/mL)					Ketoconazole (μg/mL)
	0.09375	0.1875	0.375	0.75	1.5	0.16
D101 adsorption supernatant	-	-	-	+	+	+
D101 95% ethanol eluted-fraction	-	-	-	-	+	
NKA-II adsorption supernatant	-	-	-	+	+	
NKA-II 95% ethanol eluted-fraction	-	+	+	+	+	

Note: “-” means no inhibitory effect on the normal growth of the strain; “+” means 100% inhibition rate and no growth of the strain.

**Table 2:** MIC and MBC results of different concentrations of ethanol stepwise elution samples against *Malassezia*.

Sample	Sample concentration (mg/mL)					Ketoconazole (μg/mL)	Cordycepin (mg/mL)
	0.1875	0.375	0.75	1.5	3	0.16	0.125
30% Ethanol elution fraction	-	-	+	+	+	+	+
50% Ethanol elution fraction	-	-	-	-	+		
70% Ethanol elution fraction	-	-	-	-	+		
90% Ethanol elution fraction	-	-	-	-	+		

Note: “-” means no inhibitory effect on the normal growth of the strain; “+” means 100% inhibition rate and no growth of the strain.

#### 3.2.2. Determination of nucleoside components in the 95% elution fraction of NKA-II

**Fig 3:**
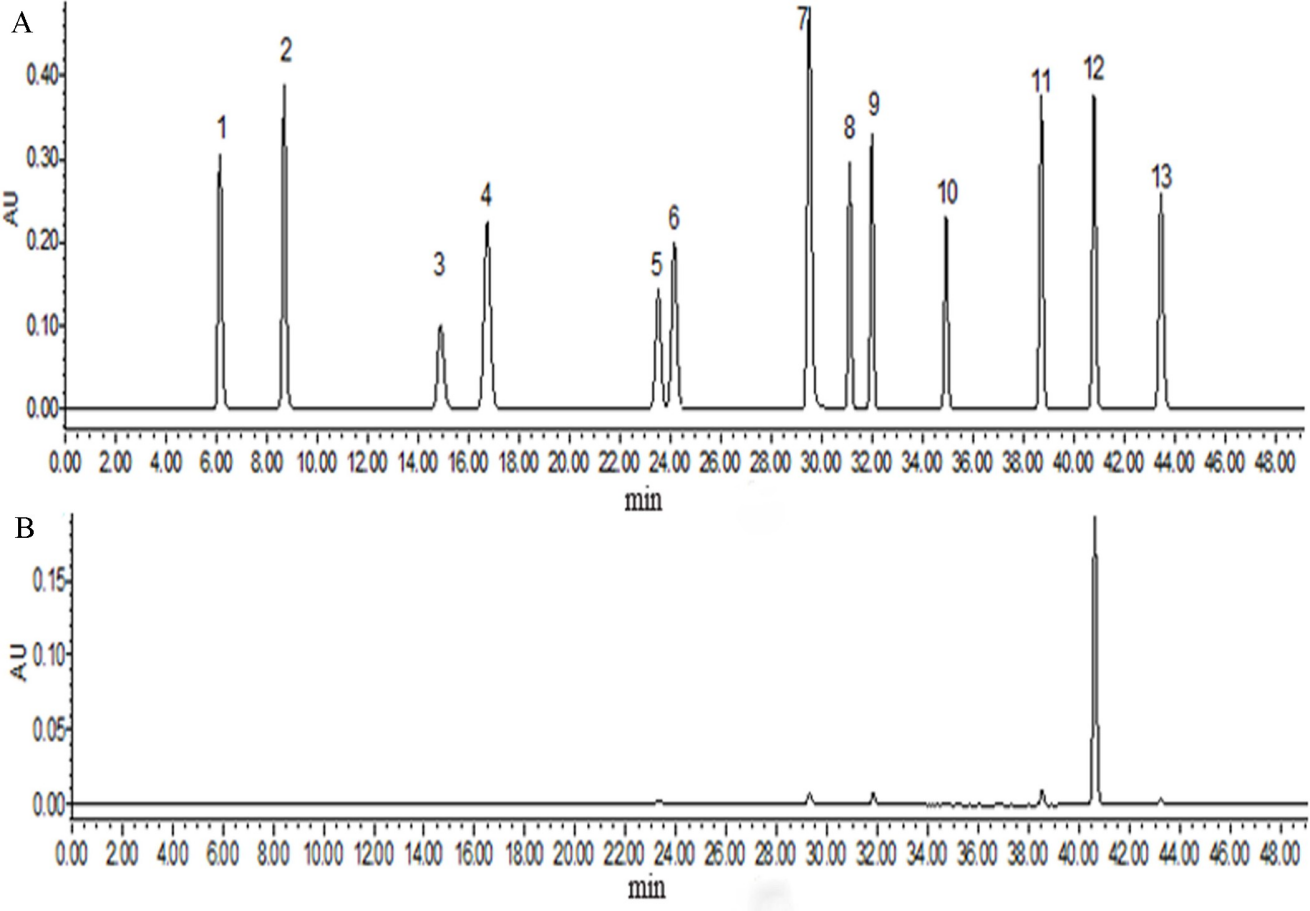
Comparison of nucleoside components in the 95% elution fraction of NKA-II with using 13 nucleoside standards. (A) HPLC chromatogram of the 13 nucleoside standards (1: cytosine, 2: uracil, 3: cytidine, 4: hypoxanthine, 5: uridine, 6: thymine, 7: adenine, 8: inosine, 9: guanosine, 10: thymidine, 11: adenosine, 12: cordycepin, and 13: N-hydroxyethyl adenosine). (B) HPLC chromatogram of the 95% eluted fraction of NKA-II.

The adsorption characteristics of NKA-II resin suggested that the inhibition component of *C*. *militaris* extract was a small molecule. Previous studies showed that the nucleosides in *C*. *militaris* have a broad-spectrum inhibitory effect on a variety of bacteria and fungi [11]. Therefore, the 95% eluted fraction of NKA-II with the highest inhibitory effect was then analyzed using 13 nucleoside standards with a standard method to determine the content of nucleosides in the fraction. The results are shown in Fig 3.

**Fig 4:**
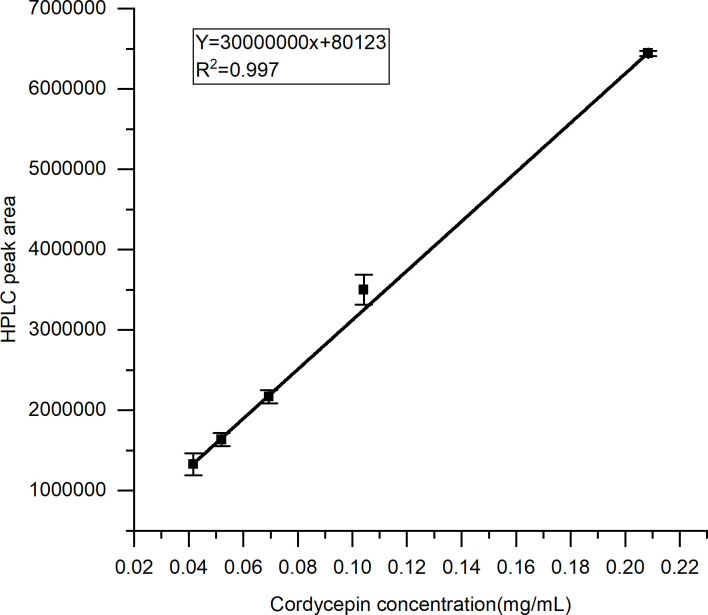
Cordycepin mass concentration and HPLC peak area standard curve.

The comparison with the mixed standards showed that the fraction contained the highest content of cordycepin, small amounts of guanosine, uridine, adenosine, N-hydroxyethyl adenosine, and adenine. Yuan et al. [25] used gradient reversed-phase HPLC for the determination of adenosine, cordycepin, cytidine, guanosine, thymidine, uridine, inosine, adenine, cytosine, thymine, uracil, and hypoxanthine in *Cordyceps militaris*. It was found that the main component of the nucleosides was cordycepin and that they also contained a small amount of uridine, adenosine and guanosine. Therefore, it was inferred that cordycepin was one of the main antimicrobial components in this fraction. To verify the correlation between cordycepin and the inhibition of *Malassezia*, the HPLC determination of the cordycepin content was performed for each sample after separation through by adsorption on NKA-II resin. The linear regression equation was similar to the results of the cordycepin standard: Y = 3 × 10^7^X+80,123; R^2^ = 0.997 (p = 0.035) (Fig 4).

#### 3.2.3. Separation of cordycepin content of each fraction through adsorption on NKA-II resin

**Fig 5:**
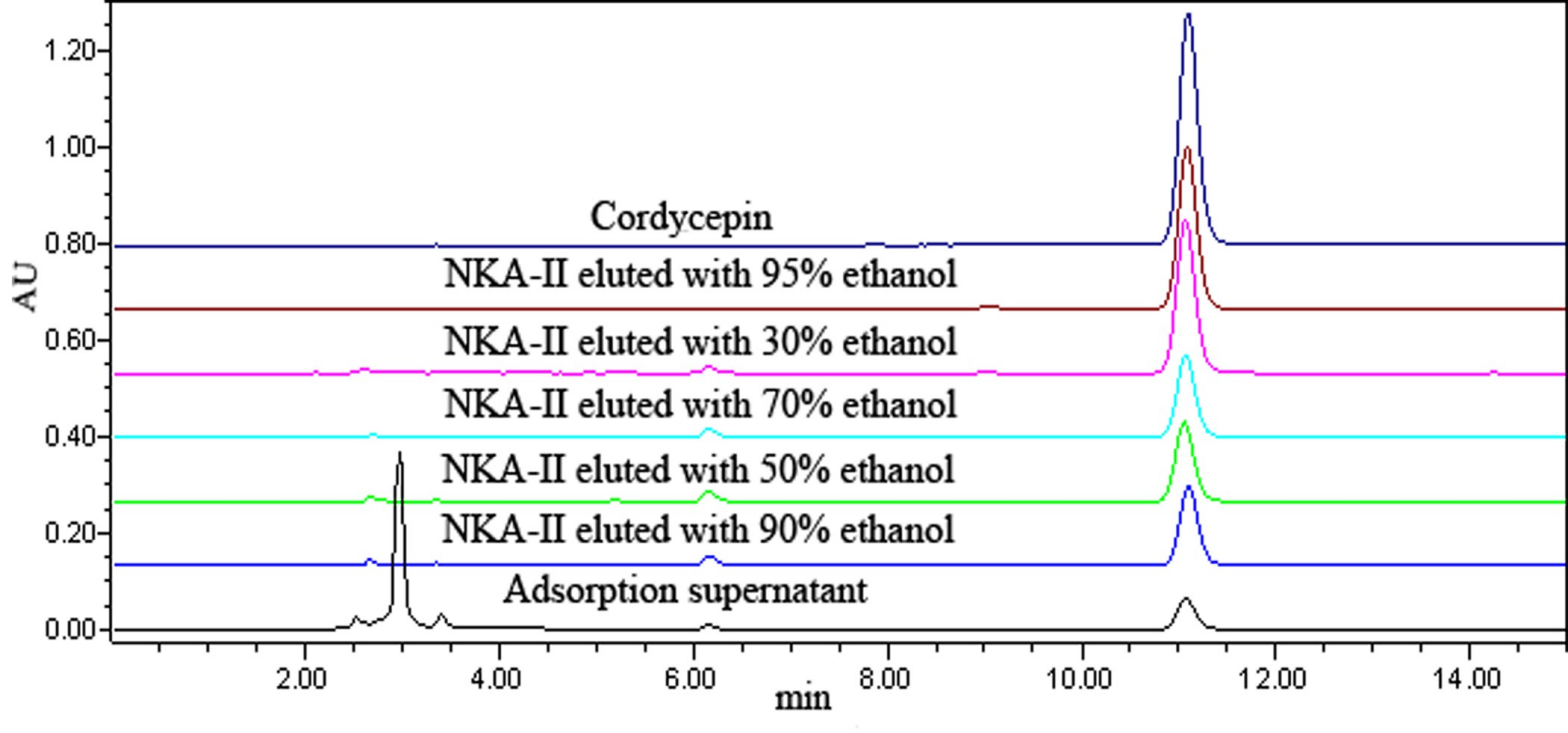
HPLC cordycepin profile of the components adsorbed and separated using NKA-II.

The cordycepin content of different fractions is shown in Table 3 and Fig 5. The 95% ethanol-eluted fraction with the highest inhibitory effect also had the highest cordycepin content (89.9%), followed by the 30% ethanol-eluted fraction with the second highest inhibitory effect and relatively lower cordycepin content (70.33%). Fractions eluted with 50%, 70%, and 90% ethanol had similarly low inhibitory effects, and similarly low cordycepin content (Table 3).

**Table 3:** Separation of the cordycepin content of each fraction through adsorption on NKA-II resin.

	Adsorption of supernatant	30% Ethanol elution fraction	50% Ethanol elution fraction	70% Ethanol elution fraction	90% Ethanol elution fraction	95% Ethanol elution fraction
Cordycepin content (%)	1.27 ± 0.02	70.33 ± 1.19**	23.45 ± 0.20**	24.13 ± 0.40**	20.71 ± 0.39**	89.90 ± 0.49**
P-value	-	7.24E-21	5.86E-15	4.07E-15	2.81E-14	3.63E-22

Note: Results are expressed as the mean ± SD (n = 3).

**: Compared with the adsorption of the supernatant, the p-value of other groups were all less than 0.01, indicating a significant difference.

(*p < 0.05; **p < 0.01).

Because the inhibitory properties of the extracts positively correlated with the content of cordycepin, the inhibitory effect of the cordycepin standard on *Malassezia* was measured and showed an MBC value of 0.125 mg/mL (Table 2), suggesting a significant inhibitory effect. In addition, it was found that the adsorption supernatant fraction with the lowest content of cordycepin was more effective than the stepwise elution fraction, indicating that the spent substrate extract of *C*. *militaris* had multiple active components working together to inhibit the proliferation of *Malassezia*, and cordycepin was one of the main inhibitory components.

Numerous studies demonstrated that many edible mushroom extracts are nutritionally safe and easily degradable sources of antimicrobial agents against human pathogens [26]. For example, the *Pleurotus florida* mycelial extract showed very effective activity against gram-positive, and gram-negative bacteria and yeast [27]. The methanolic extract of *C*. *militaris* showed strong antibacterial and antifungal properties [28]. In literature, cordycepin appeared as a key component exhibiting antibacterial activity, especially against *Escherichia coli*, *Staphylococcus aureus* [29], and *Bacillus subtilis* [30]. The highly pure cordycepin obtained using prep-HPLC demonstrated inhibitory activity against NAD+-dependent DNA ligase (LigA) from various bacteria *in vitro*. The cordycepin antibiotic could be potentially useful as a broad-spectrum antibiotic targeting LigA in a variety of bacterial species [11]. Some studies related anti-*Malassezia* activity from the extract of natural plants. Han et al. [31] found that chestnut shell and oil-soluble *Glycyrrhiza* extracts exhibited particularly high anti-*Malassezia* activity with MIC values ≤ 0.5 mg/mL, indicating that their extracts could be used as active ingredients in anti-seborrheic dermatitis and anti-dandruff shampoos. Onlom et al. [32] studied the antimicrobial activity of *Asparagus* root extracts against *Malassezia furfur*. A Saponin extract had an MIC of 0.20 mg/mL, while an ethanol extract had an MIC of 25 mg/mL, and both extracts did not interact negatively with the antifungal drugs ketoconazole and zinc azole. *Malassezia* spp. are commensal yeasts that can cause cutaneous ailments such as dandruff and seborrheic dermatitis. Thus, the spent substrate extract of *C*. *militaris* could be a safe and effective herbal treatment for various cutaneous fungal infections, including dandruff.

### 3.3. Antioxidant evaluation

**Fig 6:**
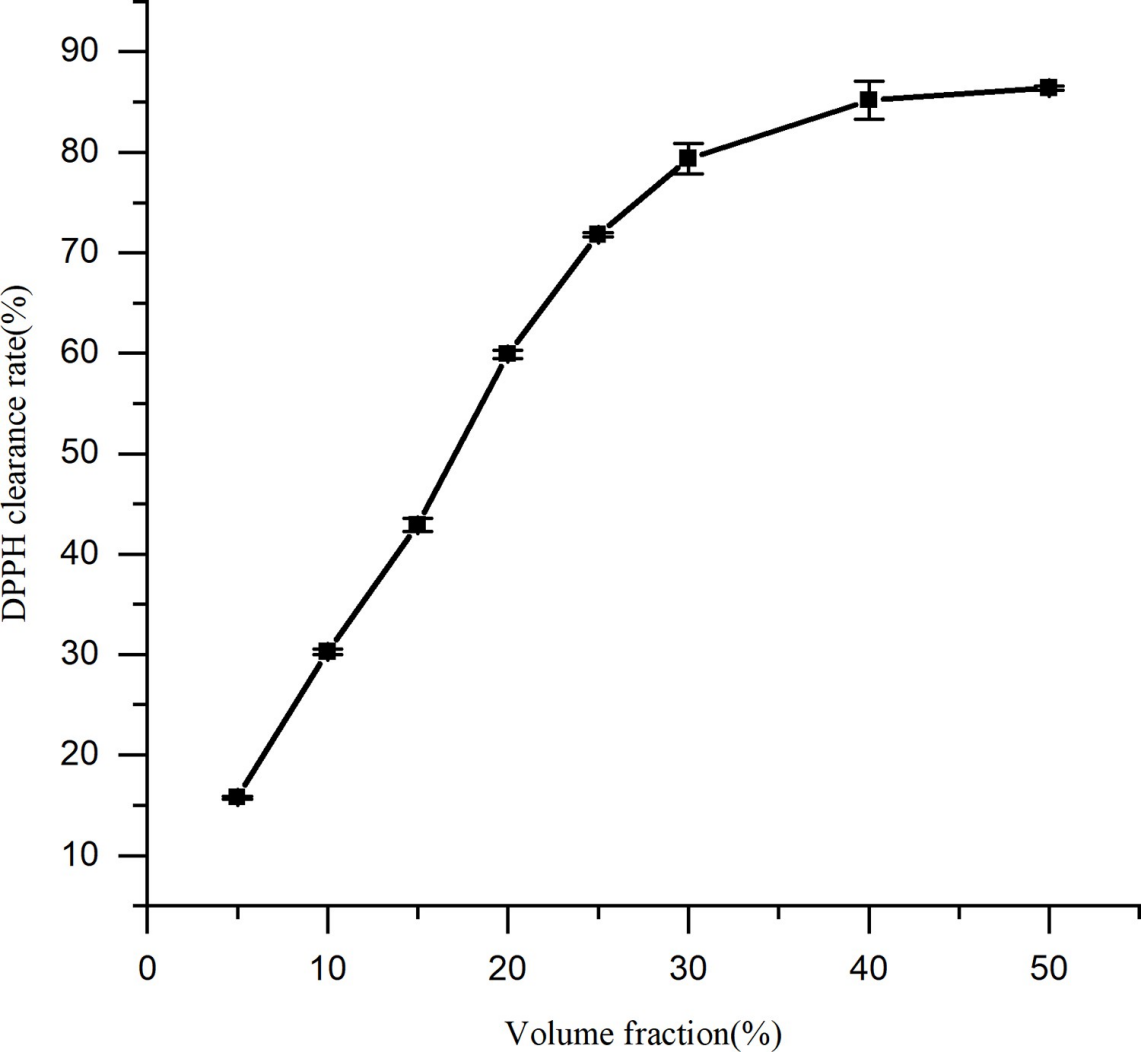
Scavenging effect of the spent substrate extract of *C*. *militaris* on DPPH. Results are expressed as the mean ± SD (n = 3).

**Fig 7:**
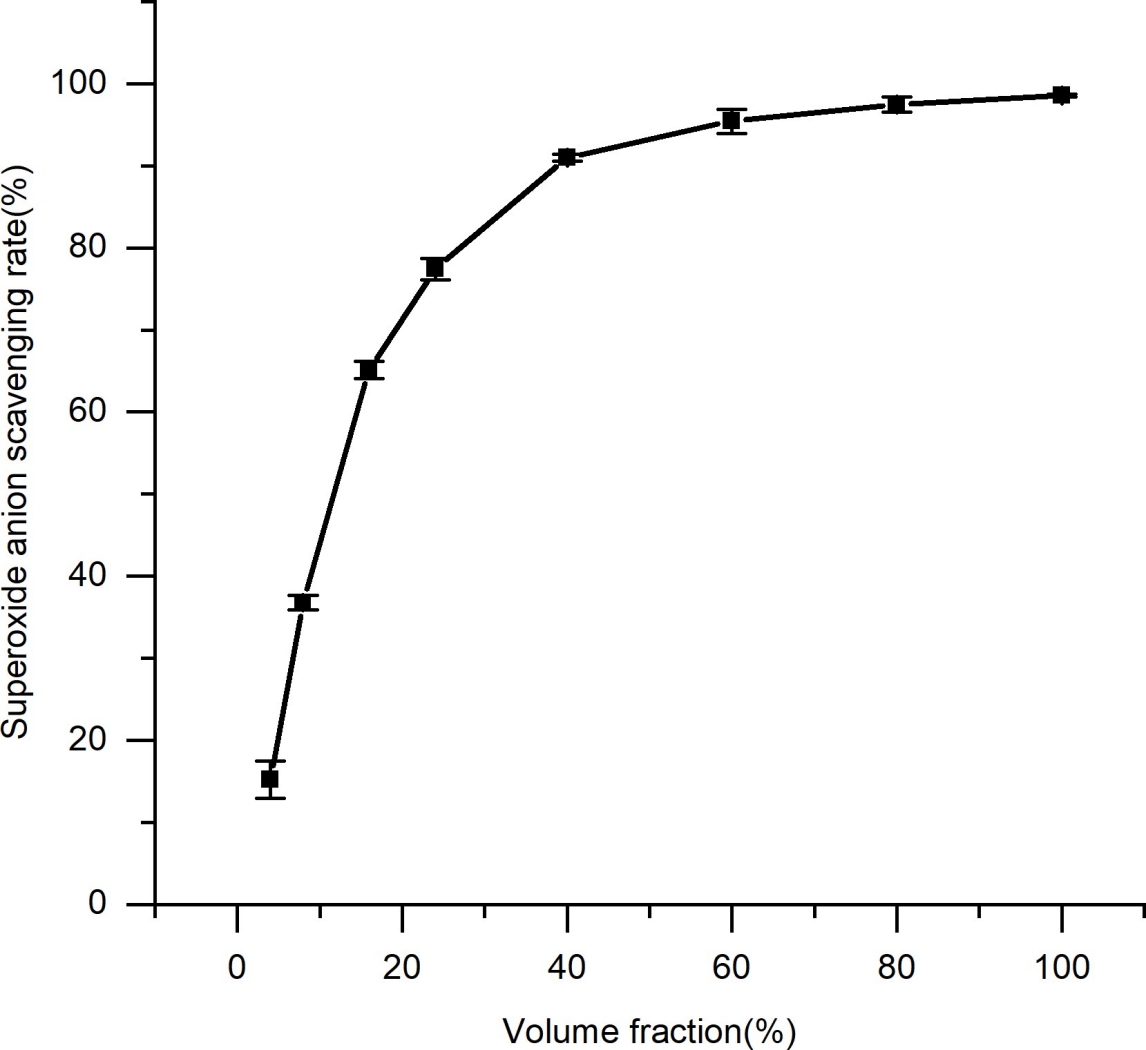
Scavenging effect of the spent substrate extract of *C*. *militaris* on superoxide anions. Results are expressed as the mean ± SD (n = 3).

**Fig 8:**
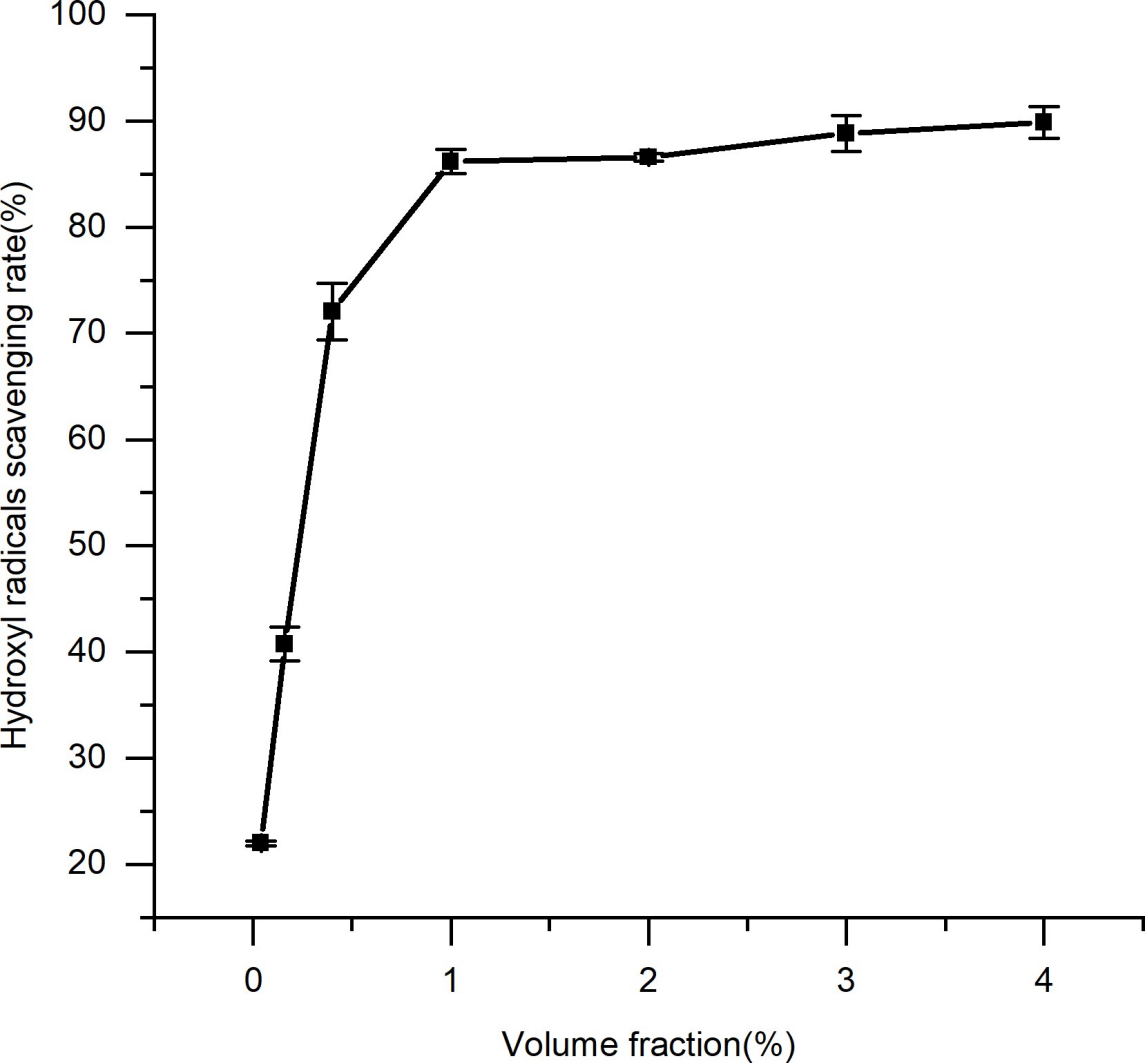
Scavenging effect of the spent substrate extract of *C*. *militaris* on hydroxyl radical. Results are expressed as the mean ± SD (n = 3).

**Fig 9:**
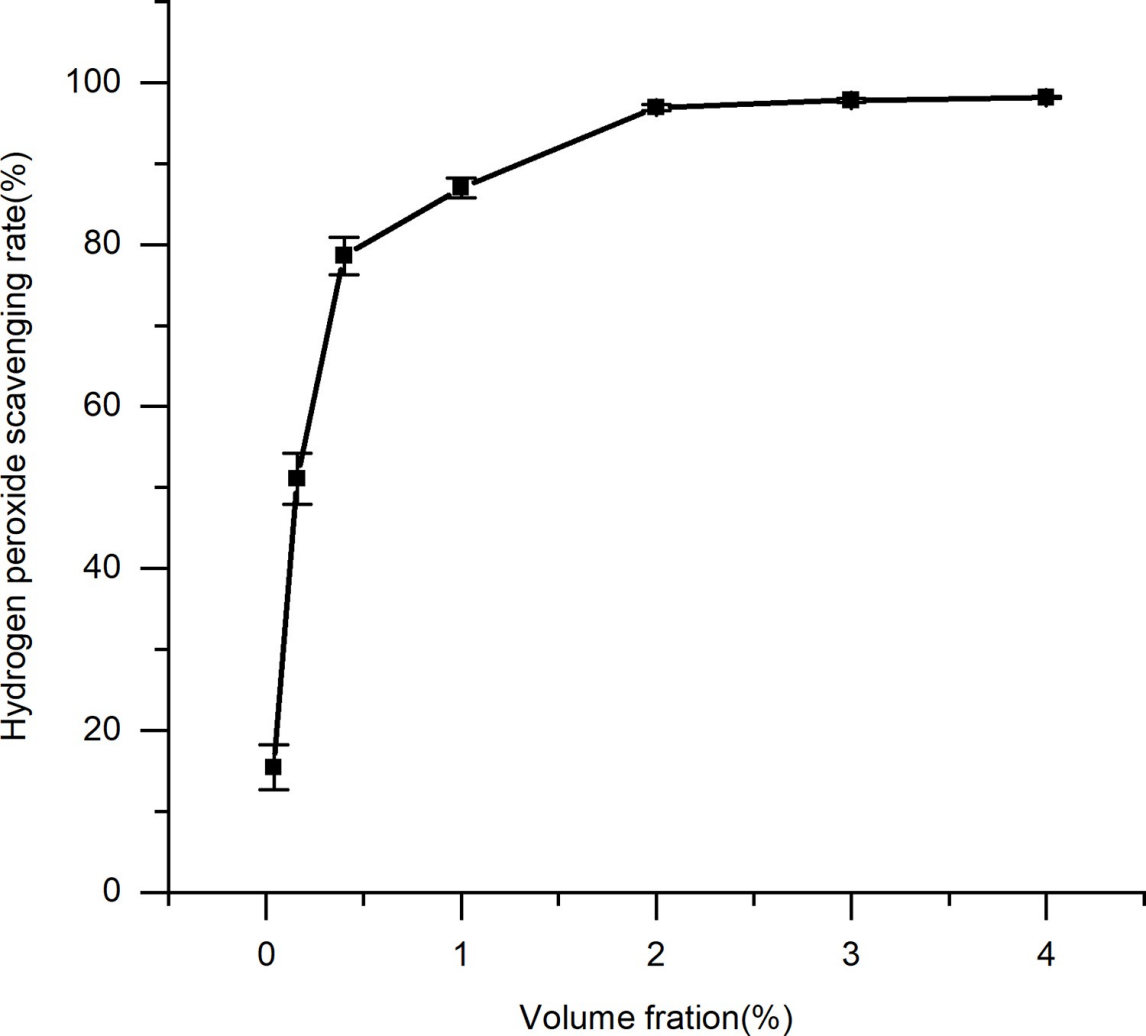
Scavenging effect of the spent substrate extract of *C*. *militaris* on hydrogen peroxide. Results are expressed as the mean ± SD (n = 3).

It was determined that the spent substrate extract of *C*. *militaris* effectively scavenged a variety of free radicals (Figs 6–9). A direct correlation between the concentration and DPPH, superoxide anion, hydroxyl radical, and hydrogen peroxide scavenging abilities was observed for the spent substrate extract of *C*. *militaris* (p = = 0.0002, 0.003, 0.0004, and 0.0006, respectively; p < 0.05). The IC50 is a half-scavenging concentration that indicates a concentration of a sample that results in a free radical scavenging rate of 50%. IC50 values of the spent substrate extract of *C*. *militaris* for DPPH, superoxide anion radicals, hydroxyl radicals, and hydrogen peroxide were 15.38%, 10.7%, 0.18% and 0.15% (w/v), respectively; converted into mg/mL, they were 3.85, 2.68, 0.05, and 0.04 mg/mL, respectively. The extract showed good scavenging abilities for all four types of free radicals, especially for the hydroxyl radical and hydrogen peroxide.

Studies have shown that free radicals damage biomolecules, causing the progression of aging, cancer, inflammation, diabetes, metabolic disorders, atherosclerosis, and cardiovascular diseases [30]. Numerous studies have demonstrated the antioxidant potential of *C*. *militaris*. The ethanol extract of *C*. *militaris* has shown antioxidant properties against DPPH, superoxide radicals, hydroxyl radicals, and low-density lipoproteins *in vitro* [33]. Zhang et al. [34] showed that polysaccharide-iron (II) extracted from *Cordyceps militaris* had significant radical scavenging activity on DPPH, hydroxyl, and superoxide species. The clearance rate of the polysaccharide-iron (II) of DPPH reached 74.02% at 2.5 mg/mL, but it had no significant effect on the clearance rate of hydroxyl and superoxide species. Compared with the polysaccharide-iron (II), our extract showed a similar scavenging ability for DPPH but stronger scavenging abilities for hydroxyl free radicals and hydrogen peroxide. Chen et al. [35] demonstrated that the polysaccharides extracted from *Cordyceps militaris* (W-CBP50) were capable of scavenging radicals such as hydroxyl, superoxide, and DPPH. The IC50 values were 1.485, 0.144 and 0.042 mg/mL. Thanh et al. [36] optimized the extraction conditions of cordycepin from *C*. *militaris* using ultrasonic-assisted enzyme extraction (UAEE) and obtained extracts with IC50 values of 0.260 ± 0.004 mg/mL for DPPH and 2.63 ± 0.22 mg/mL for H_2_O_2_. Our extract showed a greater ability to scavenge hydroxyl free radicals and hydrogen peroxide than W-CBP50 and UAEE, with IC50 values of 0.05 mg/mL and 0.04 mg/mL, respectively.

### 3.4. Effect of the spent substrate extract of *C*. *militaris* on HaCaT cells

#### 3.4.1. Effects of the spent substrate extract of *C*. *militaris* on the activity of H_2_O_2_-damaged HaCaT cells

The procedure for the evaluation of cytotoxicity initially determines the potential of already cultured cells to multiply in the presence of a test compound. As shown in Table 4, spent substrate extracts of *C*. *militaris* in the 50–60% concentration range were associated with a certain level of toxicity to HaCaT cells. Therefore, a non-toxic concentration range of approximately 2–50% was selected to determine the efficiency of the spent substrate extract in preventing H_2_O_2_ damage to HaCaT cells. There was a direct correlation between the extract concentration and the rate of cell proliferation (p = 0.033 < 0.05).

**Table 4:** Cytotoxicity of the spent substrate extract of *C*. *militaris*.

Volume fraction (%, w/v)	2	4	8	15	30	50	60
Cell proliferation rate (%)	115.78 ± 1.77	120.03 ± 1.46	96.23 ± 0.79	89.41 ± 0.56	86.12 ± 2.12	87.41 ± 2.50	61.59 ± 2.0

Note: Results are expressed as the mean ± SD (n = 3).

#### 3.4.2. Establishment of an H_2_O_2_ oxidative stress model

**Fig 10:**
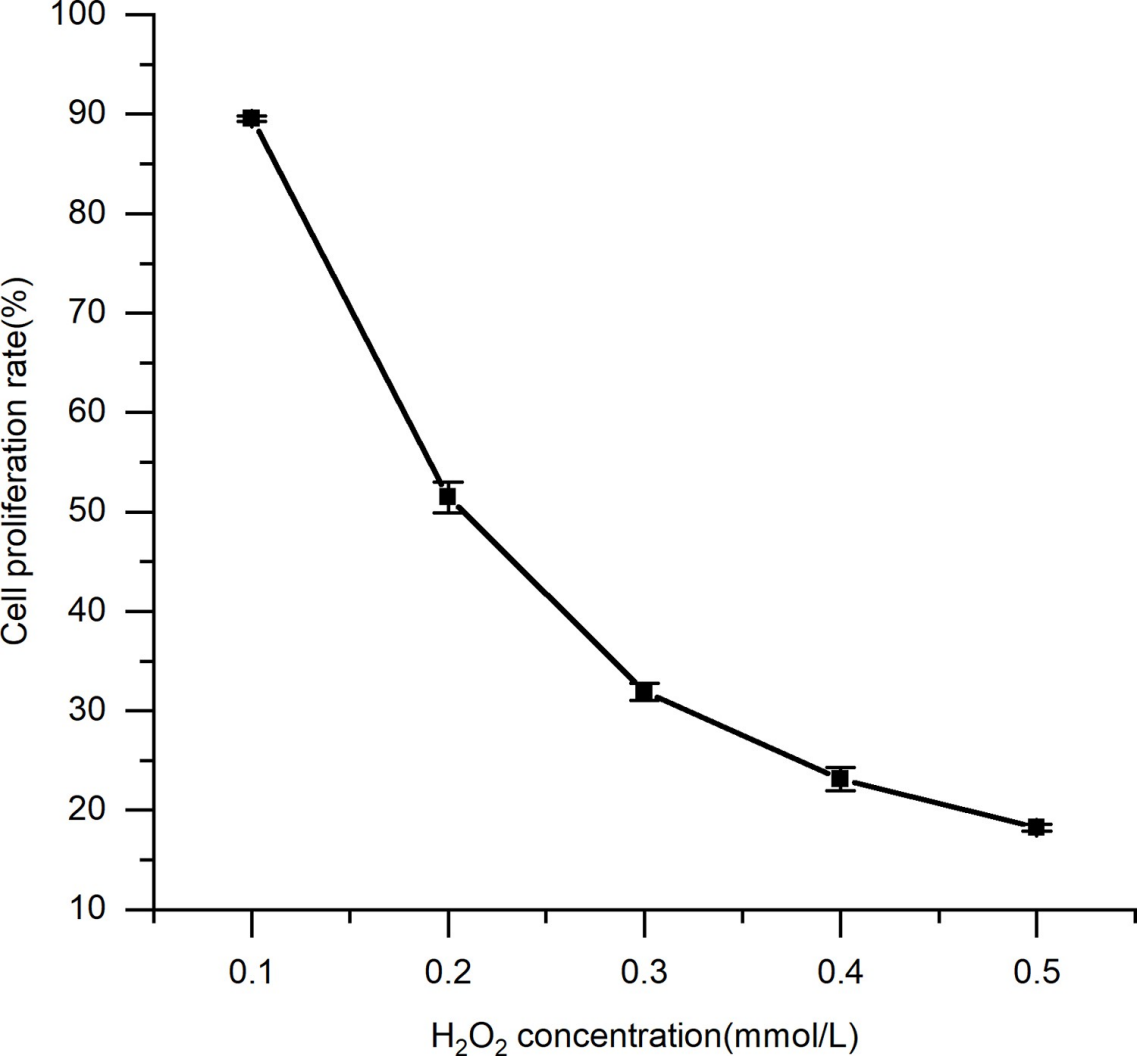
The damaging effect of H_2_O_2_ on cells. Results are expressed as the mean ± SD (n = 3).

The effect of different concentrations of H_2_O_2_ on the cell proliferation rate is shown in Table 5 and Fig 10. A cell proliferation rate of 50% was next selected as a damage model, and 0.2 mmol/L H_2_O_2_ was used to induce peroxidative damage to the cells. There was a direct correlation between H_2_O_2_ concentration and the rate of cell proliferation (p = 0.031 < 0.05).

**Table 5:** The damaging effect of H_2_O_2_ on cells.

H_2_O_2_ concentration (mmol/L)	0.1	0.2	0.3	0.4	0.5
Cell proliferation rate (%)	89.54 ± 0.29	51.46 ± 1.58	31.93 ± 0.85	23.11 ± 1.17	18.25 ± 0.31

Note: Results are expressed as the mean ± SD (n = 3).

**Fig 11:**
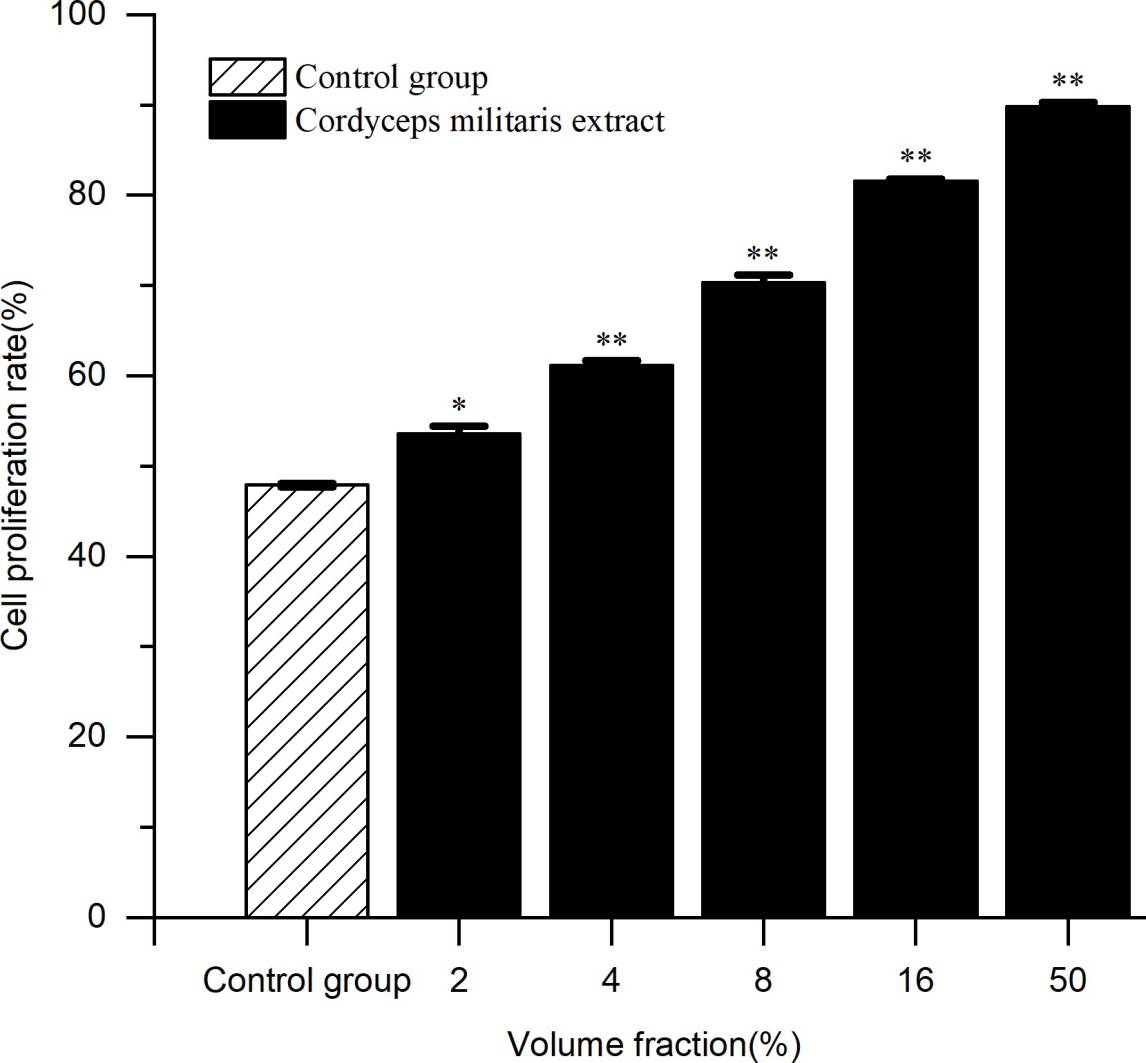
Protective effect of the spent substrate extract of *C*. *militaris* on H_2_O_2_ injured cells. Results are expressed as the mean ± SD (n = 3). *: Compared with the control group, the p-value of the extract of different concentrations were less than 0.05. (*p < 0.05; **p < 0.01).

**3.4.3. Protective effect of the spent substrate extract of *C*. *militaris* on H**_**2**_**O**_**2**_**-damaged HaCaT cells.** In the range of sample concentrations that were found safe in testing, the protective ability in terms of growth and proliferation of H_2_O_2_-damaged cells increased with increasing sample concentrations. As shown in Table 6 and Fig 11, a 50% volume fraction of extract was associated with a cell proliferation rate of 89.79 ± 0.45%.

**Table 6:** Protective effect of the spent substrate extract of *C*. *militaris* on H_2_O_2_ injured cells.

Volume fraction(%, w/v)	Control group	2	4	8	16	50
Cell proliferation rate (%)	47.91 ± 0.21	53.59 ± 0.79	61.12 ± 0.52	70.36 ± 0.74	81.56 ± 0.23	89.79 ± 0.45

Note: Results are expressed as the mean ± SD (n = 3).

H_2_O_2_ is extensively used as an indicator of oxidative stress-induced cell injury in several *in vitro* models. It was previously demonstrated that a polysaccharide derived from *C*. *militaris* protected the human hepatic cell line HL-7702 from hydrogen peroxide-stimulated apoptosis. The tested polysaccharide inhibited cell apoptosis induced by H_2_O_2_, which may have correlated with scavenging free radicals [37]. He et al. [38] investigated the anti-inflammatory properties of an ethanol extract of *C*. *militaris* based on its suppression of H_2_O_2_-related cell injury caused by Reactive Oxygen Species (ROS) production and by downregulating mitogen-activated protein kinases in C6 glial cells.

### 3.5. Anti-inflammatory capacity assay

**Fig 12:**
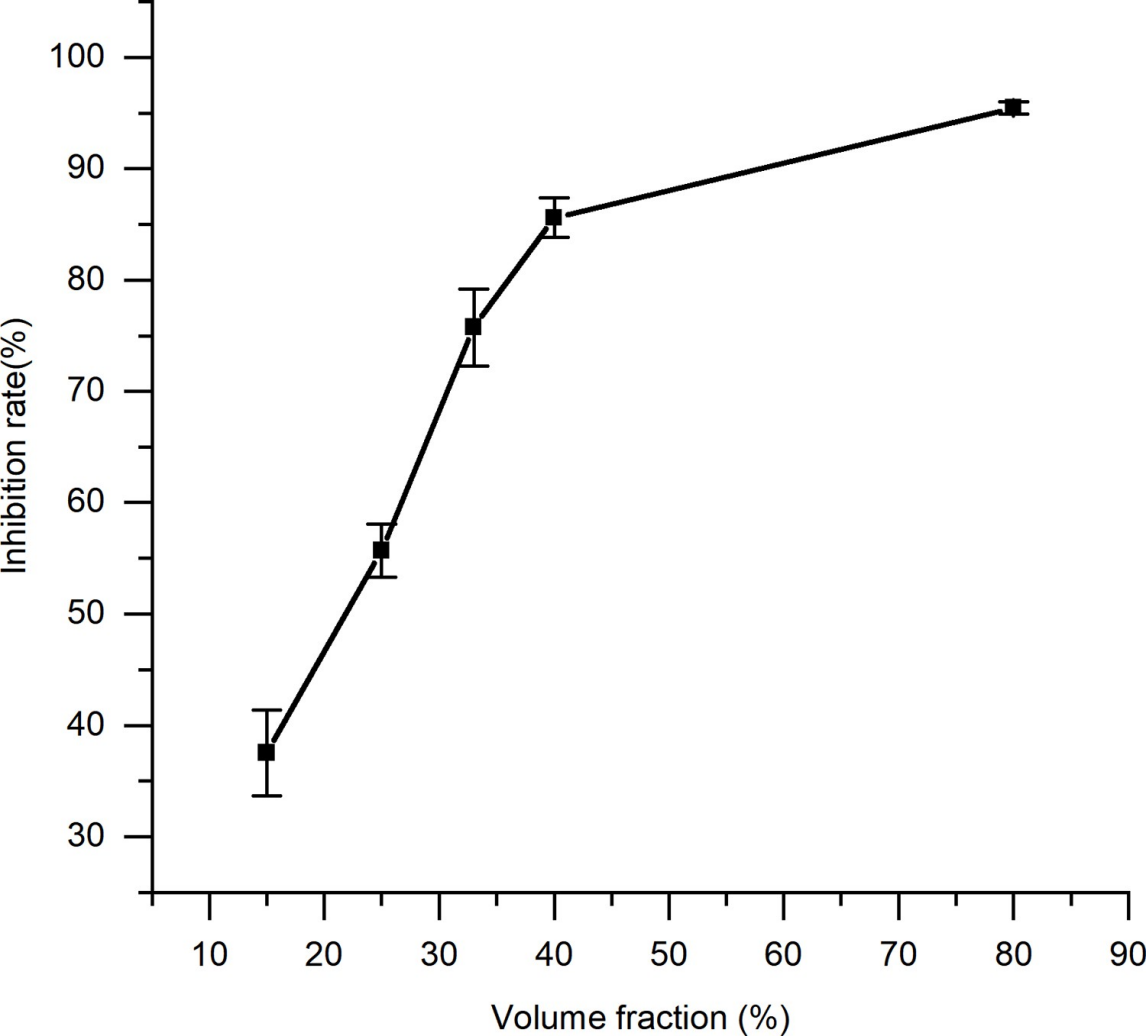
Anti-inflammatory activity of the spent substrate extract of *C*. *militaris*.

In the presence of PMA, RAW 264.7 macrophages produce large quantities of reactive oxygen species and inflammatory factors, and the resulting respiratory burst response can be measured using the luminol-dependent chemiluminescence assay. The anti-inflammatory activity of the solid fermentation extracts is shown in Table 7 and Fig 12. The blank group without PMA stimulant showed that different concentrations of the spent substrate extract of *C*. *militaris* had no effect on the autoluminescence of RAW 264.7 macrophages. In the presence of PMA, a gradual increase in the spent substrate extract concentration (10% to 90% by volume) correlated with a similar increase in the inhibition of a respiratory burst. SPSS calculations showed that the volume fraction of 40% of the extract was associated with an 85.6% respiratory burst inhibition, and a volume fraction of 20.96% resulted in 50% inhibition. A direct correlation between the concentration and the rate of respiratory burst inhibition was observed for the spent substrate extract of *C*. *militaris* (p = 0.005 < 0.05).

**Table 7:** Anti-inflammatory activity of the spent substrate extract of *C*. *militaris*.

Volume fraction(%, w/v)	15	25	33	40	80
Inhibition rate (%)	37.53 ± 3.63	55.8 ± 2.32	75.75 ± 3.46	85.60 ± 1.78	95.47 ± 0.57

Note: Results are expressed as the mean ± SD (n = 3).

Inflammation is a key function in biological processes and is initiated by various stimuli and noxious factors such as irradiation by ultraviolet light, irritants, infections, and cell injury. Currently, many researchers are interested in natural sources such as mushrooms, which contain medicinally important biofunctional components that may be able to reduce the severity of inflammatory ailments by regulating oxidative stress within physiological ranges and regulating pro-inflammatory cytokines [39]. There is both *in vitro* and *in vivo* evidence that *C*. *militaris* and its active constituents prevent inflammation in several experimental models. Cordycerebroside A, soyacerebroside I, and glucocerebroside have been isolated from *C*. *militaris* as cerebrosides (glycosphingolipids). These compounds inhibited the accumulation of pro-inflammatory iNOS protein and reduced the expression of COX-2 protein in Lipopolysaccharide-stimulated RAW 264.7 macrophages [40]. Recent studies showed that cordycepin, one of the prominent components of *C*. *militaris*, inhibited inflammation-associated expression of the genes encoding COX-2 and iNOS [41]. Lei et al. [42] demonstrated that cordycepin suppressed LPS-induced malondialdehyde (MDA) content and inflammatory cytokine (IL-1β and TNF-α) production. Maya et al. [43] used membrane stabilization of human red blood cells (HRBCs) and were able to show and to quantify anti-inflammatory activity in plant extracts. Their experiment revealed that at a dose of 500 μg/mL, the anti-inflammatory activity of a hot water extract of *Wattakaka volubilis* leaves was 54.27%, as indicated by its ability to protect HRBC membranes from hyposaline-induced lysis. *C*. *militaris* and its bioactive components are being investigated for biomedical applications due to their minimal side effects, high nutritional content, and suppression of inflammation.

## 4. Conclusions

The present study highlighted that the spent substrate leftovers from the cultivation of *C*. *militaris* can potentially be used as a new material source for the preparation of crude extracts for use in pharmaceutical or cosmetic applications. The results obtained from this study revealed that the spent substrate extract of *C*. *militaris* has a certain inhibitory effect on *Malassezia* and that cordycepin was the main antimicrobial active component in the extract.The results of this study also indicated that the extract has strong antioxidant and certain anti-inflammatory activities, can reduce oxidative damage and inhibit the subsequent inflammatory response to a certain extent, and is non-toxic to cells in the range of 2–50%. These results suggested that the extract could be potentially used to develop safe and effective functional medicines and cosmetics for cutaneous fungal infections. Our data strengthens a need for further research into the spent substrate of *C*. *militaris* for its therapeutic and repair uses.

## Supporting information

S1 Raw data Cordycepin content. (XLSX)

S2 Raw data DPPH, O2−, -OH, and H_2_O_2_. (XLSX)

S3 Raw data Cytotoxic activities. (XLSX)

S4 Raw data Anti-inflammatory activities. (XLSX)
